# Biodegradable Antimicrobial Films for Food Packaging: Effect of Antimicrobials on Degradation

**DOI:** 10.3390/foods10061256

**Published:** 2021-06-01

**Authors:** Eva Hernández-García, María Vargas, Chelo González-Martínez, Amparo Chiralt

**Affiliations:** Instituto Universitario de Ingeniería de Alimentos para el Desarrollo, Universitat Politècnica de València, 46022 València, Spain; evherga1@upvnet.upv.es (E.H.-G.); cgonza@tal.upv.es (C.G.-M.); dchiralt@tal.upv.es (A.C.)

**Keywords:** biopolymer, active packaging, biodegradation, composting, antimicrobial

## Abstract

The environmental problem generated by the massive consumption of plastics makes necessary the developing of biodegradable antimicrobial materials that can extend food shelf-life without having a negative impact on the environment. The current situation regarding the availability of biodegradable food packaging materials has been analysed, as well as different studies where antimicrobial compounds have been incorporated into the polymer matrix to control the growth of pathogenic or spoilage bacteria. Thus, the antimicrobial activity of active films based on different biodegradable polymers and antimicrobial compounds has been discussed. Likewise, relevant information on biodegradation studies carried out with different biopolymers in different environments (compost, soil, aquatic), and the effect of some antimicrobials on this behavior, are reviewed. In most of the studies, no relevant effect of the incorporated antimicrobials on the degradation of the polymer were observed, but some antimicrobials can delay the process. The changes in biodegradation pattern due to the presence of the antimicrobial are attributed to its influence on the microorganism population responsible for the process. More studies are required to know the specific influence of the antimicrobial compounds on the biodegradation behavior of polymers in different environments. No studies have been carried out or marine media to this end.

## 1. Introduction

The food industry is responsible for a high consumption of plastics for food packaging, which is essential to maintain food safety. Traditionally, food companies have packaged their products in metal and glass containers, but nowadays the use of plastic containers to pack food has become global [1] due to the great advantages of plastic. It is light, with versatile mechanical and optical properties, moldable, impermeable to water and gases, resistant to corrosive chemicals, with low density and low cost, and allows for the printing of relevant information for the consumer. Moreover, plastic-based packaging materials favor the preservation of food through the application of different strategies such as the incorporation of antimicrobials and antioxidants or the development of modified atmospheres. However, a great part of synthetic plastics generates a large amount of waste that decomposes very slowly, accumulating in terrestrial and marine ecosystems, causing the known great environmental problem. In addition to the physical impacts on terrestrial and marine ecosystems, there is growing concern about the impact on human health because of the toxic substances (flame retardants, pigments, plasticizers, compatibilizers, etc.) used in plastic fabrication, which can migrate to water or other contact media including food. Likewise, one of the main problems is microplastics that, unlike larger plastics, are not easily seen at naked eye and once these particles end up in the ocean, their recovery is no longer possible. Microplastics enhance the transport and bioavailability of toxic, bio-accumulative and persistent organic pollutants (POPs) that could enter the food chain through consumption of marine products [2].

In recent years, the greater environmental awareness of citizens and the new European regulations on this issue, has given rise to the new concept of sustainable packaging. Thus, research in renewable raw materials, biotransformation process, structural design and biodegradability has been extended. In this sense, the interest in the so-called bioplastics has rose with new developments that provide an alternative to traditional polymers [3]. Bioplastics are polymers that come from renewable natural sources or are biodegradable, or both, such as starch or cellulose [4]. The global production of bioplastic was 2.1 million tons in 2018, with prevision of 2.4 million tons for 2024. From that, only about 55% are biodegradable, the main polymers being poly(butylene adipate-co-terephthalate): PBAT (13.4%), Polybutylene succinate: PBS (4.3%), polylactic acid: PLA (13.9%), polyhydroxyalkanoates: PHAs (1.2%) and starch blends (21.3%) [5]. An increase in the bioplastic production as well as the adequacy of the properties of for determined target applications are necessary to promote the sustainable use of plastics.

The development of biodegradable and sustainable packaging also requires adding other substances to the polymer matrix to adapt its properties to the specific needs, which could affect biodegradation behavior. Plasticizers are used to improve the mechanical performance of the polymer matrix since they weaken the cohesion forces between polymer chains, increasing their mobility and improving the flexibility of the polymer matrix. Polyols, such as glycerol, polyethylene glycol, propylene glycol, sorbitol, sucrose or glucose are commonly used plasticizers in the formulations based on hydrophilic polymers. Other plasticizers of hydrophobic nature can also be used, such as fatty acids and their derivatives and oils [6].

Biodegradable food packaging materials can include antimicrobial and/or antioxidant components that improve their performance, extending the shelf life of the packaged food. The incorporated antimicrobial agents prevent the undesirable growth of microorganisms on the surface of the food more efficiently than their direct incorporation into the food [7], due to their controlled-release from the packaging to the product. The controlled release can extend the antimicrobial action over time in a more constant way, thus requiring lower doses of active compounds [8]. However, the presence of antimicrobial compounds in the material could seriously affect the biodegradation pattern of the carrying bioplastic by interfering the action of the natural microbial population responsible for the biodegradation process.

The aim of this work was to analyze different studies developing antimicrobial materials based on biodegradable polymers, as well as the influence of the incorporation of these compounds on the biodegradation behavior of the active materials.

## 2. Antimicrobial Packaging Materials Based on Biodegradable Polymers

**Figure 1:**
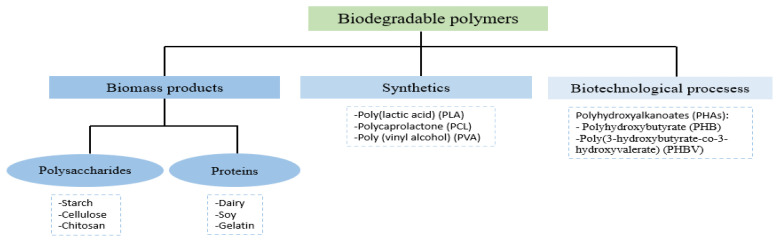
Classification of biodegradable polymers according to their origin.

Biodegradable polymers for developing active packaging are classified in three main groups, as described in Figure 1 [9]. The first group corresponds to those obtained from biomass, such as the biopolymers extracted from agro-food resources or waste such as polysaccharides (starch, cellulose or chitosan) and proteins (dairy and soy proteins or gelatin). The second group corresponds to synthetic polymers obtained from monomers from renewable sources (such as PLA), or from oil (such as polycaprolactone: PCL or poly (vinyl alcohol): PVA). The third group consists of polymers produced by microorganisms, obtained from biotechnological processes through the extraction of cultures, such as PHAs.

Different studies have been carried out to obtain antimicrobial packaging materials by incorporating active components into biodegradable polymer matrices, as commented below.

### 2.1. Polymers from Biomass

Starch is the biopolymer obtained from biomass that shows the highest production and number of studies due to its high availability, low cost and suitable properties for food contact. As shown in Table 1, numerous studies have been carried out on the development of active films with starch from different sources incorporating antimicrobials. In most cases, these films were obtained by casting, although some authors have also reported interesting results for thermo-processed films with broader industrial applications [10,11,12]. Different authors incorporated essential oils as antimicrobial agents in starch matrices to obtain active films, as can be seen in Table 1.

Silveira et al. [13] incorporated tea tree essential oil (0.08, 0.8, and 1.5% *v*/*v*) with cellulose nanofibers by casting technique, obtaining notable growth inhibition for *S. aureus* (73%) and *C. albicans* (63%), but without significant inhibition of the growth of *E. coli* in *in vitro* tests. Dhumal et al. [21] incorporated carvacrol (0.75% *w*/*w*) and citral (1.0% *w*/*w*) and both (0.75% carvacrol and 1.0% citral) in sago starch films with guar gum, prepared by casting, which exhibited good antimicrobial activity against *B. cereus* and *E. coli* in *in vitro* tests. The Petri plates were placed at 40 °C for sago starch/guar gum films and 26 °C for sago starch/guar gum/essential oil films. Valencia-Sullca et al. [10] obtained starch-chitosan bilayer films by thermo-compression, either containing essential oils (oregano or cinnamon leaf at 0.25% *w*/*w*) of not, incorporated into the chitosan monolayer that was obtained by casting. These bilayers were effective at controlling the bacterial growth in coated pork meat samples, but the thermal treatment used to form the bilayers reduced the antimicrobial effect of chitosan as compared to the chitosan monolayers. Moreover, the incorporation of essential oils into the chitosan layer did not improve the antimicrobial action of the films. Syafiq et al. [17] obtained films with palm sugar starch and palm sugar nanocrystalline cellulose, incorporating cinnamon essential oil (0.8, 1.2, 1.6 and 2.0 wt%) by casting, which showed inhibition of *B. subtilis*, *S. aureus* and *E. coli* growth in *in vitro* tests. Cano et al. [19] obtained starch/PVA films containing neem (NO) or oregano essential (OEO) oil. These were incorporated into the films, obtained by casting, at two different ratios with respect to the starch, 1:0.125 (S-PVA-1OEO and S-PVA-1NO) and 1:0.5 (S-PVA-2OEO, S-PVA-2NO). The films exhibited antibacterial effect against *L. innocua* and *E. coli*, and antifungal properties against *A. niger* and *P. expansum*). Perdana et al. [20] developed starch-based composite films and coatings with lemongrass, obtained by casting. Lemongrass was added separately to obtain 1%, 1.5% and 2% of concentration to the film-forming solution of cassava starch (3% *w*/*v*). The coatings were effective at reducing mesophilic bacteria count and fungi count in cold-stored chilies. Other active compounds, such as sodium dehydroacetate and rosemary extract [22], silver nanoparticles [15], pomegranate peel [26], chitosan [10,16,18], N-α-lauroyl-l-arginine ethyl ester monohydrochloride [12] or sodium benzoate and citric acid [24] have also been incorporated into starch-based matrices for the purposes of obtaining antimicrobial packaging materials. Yan et al. [22] incorporated sodium dehydroacetate and rosemary extract to oxidized and acetylated corn starch films with sodium alginate that were prepared by casting. Sodium dehydroacetate was added to reach a final content of 0, 0.1, 0.3, 0.5 and 0.7% (*w*/*w*), and rosemary extract was added at a concentration of 0, 0.3, 0.6, 0.9 and 1.2% (*w*/*w*). All films showed antimicrobial effect against *E. coli*, whereas an effective inhibition of Aspergillus niger was only observed when sodium dehydroacetate was incorporated in the films. Ali et al. [26] incorporated pomegranate peel powder at different concentration based on the dry starch content (0, 2, 4, 6, 8, 10, 12, and 14 wt%) in high-amylose hydroxypropylated starch films plasticized with glycerol and observed the growth inhibition of both gram-positive (*S. aureus*) and gram-negative (Salmonella) bacteria in *in vitro* tests; the greatest effect being observed against *S. aureus*. Valencia-Sullca et al. [11] obtained cassava starch-chitosan films by melt-bending and compression moulding, using glycerol and polyethylene glycol as plasticizers. The incorporation of the highest amount of chitosan (70:30 starch chitosan ratio) in the films led to the reduction in coliforms and total aerobic counts of cold-stored pork meat slices, thus extending their shelf-life. Hasan et al. [16] developed films based on brown rice starch and chitosan (starch/chitosan ratio: 70:30, 50:50 and 30:70), plasticized with palm oil and prepared by casting. Films showed a remarkable antimicrobial *in vitro* effect against gram-positive bacteria (*S. aureus*) and gram-negative bacteria (*E. coli*). Shapii’ et al. [18] obtained tapioca starch/chitosan films with different concentration of chitosan nanoparticles (0, 5, 10, 15, 20% *w*/*w*), which showed bacterial growth inhibition both in *in vitro* tests (*B. cereus*, *S. aureus*, *E. coli* and *S. typhimurium*) and when the films were applied to wrapped cherry tomatoes. Moreno et al. [12] developed films based on corn starch and bovine gelatin with N-α-lauroyl-l-arginine ethyl ester monohydrochloride (10 wt%) that were capable of extending the shelf-life of chicken breast fillets, without affecting meat oxidation. These films were also effective at reducing the total viable counts, which remained below the legal limit, in marinated salmon samples stored for 45 at 5 °C [25]. De Moraes et al. [24] developed starch films with sodium benzoate (0.001 g/100 g of tapioca flour), citric acid (30 g/100 g tapioca flour) and the mixture of both, which reduced the growth of *L. innocua* in inoculated Cheddar cheese samples.

### 2.2. Synthetic Polymers

In this group PVA and polyesters, such as PLA and PCL are included. PLA is a thermoplastic biopolymer obtained from lactic acid by the starch fermentation. Due to its biodegradability in a compost medium (compostability) and its biocompatibility, PLA has found numerous applications since it also shows good barrier properties against water vapor, O_2_ and CO_2_, high mechanical resistance and photostability [27]. PVA is a highly hydrophilic polymer obtained through the hydrolysis of poly (vinyl acetate) [28,29]. PCL was first synthesized in the 1930s by the ring-opening polymerization of ε-caprolactone and it is highly hydrophobic with longer degradation times than PLA [30], making it suitable for applications where long degradation times are required. Due to its low melting temperature, PCL is easily processed using conventional melting techniques. Its mechanical properties can be improved by different fillers (particles or fibers) [31]. Although it is a biodegradable material, PCL comes from non-renewable petrochemical sources and is frequently used as a copolymer with PLA to prepare degradable blends with specific properties [31].

Different studies have been carried out aimed to obtain antimicrobial packaging materials based on PLA, PVA or PCL (Table 2).

Ahmed et al. [32] developed films based on PLA with polyethylene glycol (PEG) and essential oils of cinnamon (0.4, 0.8, 1.2 and 1.6 mL were poured to the PLA/PEG solution), garlic (1.6 mL in the PLA/PEG solution) or clove (1.6 mL in the PLA/PEG solution) that were obtained by casting. These films showed antimicrobial activity against different bacteria. *C. jejuni* showed greater sensitivity (7 log reduction) than *S. aureus* (2 log reduction) to cinnamon and clove oils incorporated into PLA films. Garlic oil incorporated into the PLA films exhibited limited antimicrobial activity against both bacteria. Khodayari et al. [35] developed PLA films with an ethanolic extract of propolis (0, 1 and 2% *v*/*v*) and essential oil of *Tanacetum balsamita* (0, 1 and 2% *v*/*v*), which were able to control the growth of gram-positive and gram-negative bacteria. The *in vitro* results showed that the ethanolic extract of propolis had no significant antimicrobial activity, but when the extract was added in combination with the essential oil of *Tanacetum balsamita* an effective antimicrobial effect was achieved. The gram-positive bacteria were more sensitive than the gram-negative, especially, *B. cereus*, which was the most sensitive to Tanacetum balsamita essential oil. Furthermore, the same authors studied the antimicrobial effect of these films on precooked sausages. During cooking, the counts of heat-sensitive bacteria, such as *Enterobacteriaceae* or *S. aureus*, dropped below the limit of detection. However, psychrotrophic bacteria went from being below the detection limit on day 0 to a detectable level, below the permissible limit after 50 days of storage, evidencing the protective effect of the films. Jiang et al. [33] developed films of PLA, PBAT (poly(butylene adipate-co-terephthalate)) and cellulose silver nanocrystals (0 to 8 wt% based on the total weight of the PLA/ PBAT component), which were prepared by casting and were effective at inhibiting the growth of *E. coli* and *S. aureus*. The film without the cellulose-silver nanoparticles did not show any inhibitory effect. Coltelli et al. [34] developed PLA and PBS (poly(butylene succinate)) films with chitin nanofibrils (2 wt%) used as filler material. The antimicrobial tests did not revealed effectiveness against *S. aureus* and *Enterobacter* spp. Muller et al. [36] incorporated cinnamaldehyde (CIN) in PLA films (PLA:CIN ratio of 10:2.5) to obtain starch-PLA active bilayers. The PLA monolayers exhibited antibacterial effect against *E. coli* and *L. innocua*, whereas the bilayers were more effective when the starch side was in contact with the culture medium. Extruded PLA films with different amounts of chitosan powder were effective at controlling the growth of total aerobes and coliforms in meat, especially when the particle size of chitosan was more reduced [37].

Olewnik-Kruszkowska et al. [38] developed films composed of PVA and chitosan (Ch) with the addition of poly- (hexamethylene-guanidine) (PHMG) (0.5 or 1 wt% of the PVA or PVA:Ch polymer mass) that were obtained by casting. This study confirmed the biocidal potential of PVA films with PHMG, showing their antimicrobial potential against gram-positive (*S. aureus*) and gram-negative (*E. coli*) bacteria. Haghighi et al. [39] developed films of chitosan-PVA mixtures with different concentrations of lauroyl ethyl arginate (LAE) (1, 2.5, 5 and 10 % *w*/*w* of biopolymer). Films were obtained by casting and drying at 25 ± 2 °C overnight. These films inhibited the growth of four foodborne bacterial pathogens, *Campylobacter jejuni*, *Salmonella typhimurium*, *Escherichia coli*, and *Listeria monocytogenes*, being the films with a content of 5 to 10% of the antimicrobial the most effective. Suganthi et al. [40] used organic acids (tartaric, lactic and malic) as crosslinking agents in PVA films (10 wt% of acid in proportion to PVA) that were obtained by casting. The films containing lactic acid exhibited the highest bacterial inhibition, which was largely attributed to its ability to modify the local pH and alter the permeability of the microbial membrane, interrupting the bacterial-substrate interaction. Tripathi et al. [41] developed films based on PVA and chitosan, which was previously dissolved into 2 wt% acetic acid to prepare a 1 wt% chitosan solution and evaluated their antimicrobial effect on minimally processed tomato. The antimicrobial films were obtained by mixing chitosan and PVA with glutaraldehyde as a crosslinking agent. A greater growth inhibitory effect was observed in *E. coli* and *B. subtilis* as compared to *S. aureus*.

As regards active films based on PCL, Li et al. [42] developed an active film using electrospun membranes composed of PCL and a short-chain peptide called REDV, with eugenol as antimicrobial agent (5, 10, 20 and 30 wt%). Eugenol films were effective against gram-positive bacteria, such as *S. aureus* and gram-negative bacteria, such as *E. coli*. Salević et al. [43] produced PCL films with sage extract (5%, 10%, and 20% *w*/*w* with respect to the polymer content) incorporated as an antimicrobial agent and using the electrospinning technique followed by annealing treatments. The films were effective against gram-negative bacteria (*E. coli*) and gram-positive bacteria (*S. aureus*), being more effective against the gram-positive. Takala et al. [44] prepared trilayer bioactive films with methylcellulose (MC) and PCL. Two antimicrobial formulations named A (60 g/L organic acids mixture, rosmarinic acid extract and 6 g/L Asian essential oil blend) and B (60 g/L organic acids mixture, rosmarinic acid extract and 6 g/L Italian essential oil blend) were added to the MC films during melt blending, and the three-layer films (PCL/MC/PCL) were obtained by compression molding. These films were applied to broccoli samples that were stored at 4 °C for 12 days. The films significantly reduced the growth of *Escherichia coli* in the broccoli samples from day 4, and there was a total inhibition on day 12. Similarly, a significant reduction in Salmonella typhimurium counts was observed from 2 days and a total inhibition on day 7. Lyu et al. [45] also conducted a study with PCL composite films with different concentrations of grapefruit seed extract (GSE) (0, 1, 3 and 5 %) added as antimicrobial agent. The antimicrobial activity of the films increased as the concentration of GSE increased; with a greater inhibitory activity against *Listeria monocytogenes* reported for the films that incorporate 5% GSE. When these films were applied to commercial cheddar cheese packages, a delay in microbial growth of the samples was observed. Mixed PCL/starch/pomegranate rind powder films (5, 10, 15, 20, 30 and 40 % of pomegranate rind powder) were also developed for antimicrobial packaging, observing antimicrobial effectiveness for high concentrations of pomegranate rind (40%) [46]. Tampau et al. [48] developed three-layer starch films with carvacrol (15 wt% with respect to the polymer), which were loaded in electrospun PCL fibers placed between the two starch sheets. *In vitro* microbiological tests did not show growth inhibition of *Listeria innocua*, but the films were effective against *E. coli*. The total carvacrol load in the three-layer determined the film effectiveness depending on the minimal inhibitory concentration of the bacteria.

### 2.3. Polymers from Microorganisms

Among the polymers obtained by fermentation, PHAs are linear polyesters produced by bacteria by the fermentation of sugars or lipids. They are produced by a wide variety of bacteria throughout a carbon and energy storage mechanism. Thus, PHAs can be synthesized from renewable carbon sources, are biodegradable (they can be assimilated by many microorganisms, either from soils, seas, lakes or sewage) and are biocompatible (without toxic effects). These properties make PHAs very interesting as substitutes for conventional plastics such as PP and PET with similar physical characteristics [49].

The two most common PHAs are polyhydroxybutyrate (PHB) and poly(3-hydroxybutyrate-co-3-hydroxyvalerate) (PHBV). PHB is a crystalline, biodegradable polyester with a melting temperature between 173 °C and 180 °C, close to that of PLA, which facilitates their blending in order to modulate the properties. PHB is a relatively rigid and fragile bioplastic and has a low resistance to thermal degradation, which limits its thermoprocessing [50]. PHBV contains units of 3-hydroxyvalerate (HV) inserted in the PHB polymer. PHBV is an aliphatic polyester, non-toxic, 100% biodegradable and biocompatible with many types of cells. PHBV is characterized by its high degree of crystallinity and resistance to ultraviolet radiation and acceptable amounts of alcohols, fats and oils. However, it is a rigid and quite fragile polymer, with a melting temperature of 153 °C, which is lower than that of PHB. PHBV has high viscosity in liquid state (which favors the extrusion processes) and its films show good barrier capacity to oxygen, and better mechanical properties and greater flexibility than PHB films. Despite some of the improvements that PHBV offers over PHB, this polymer continues to exhibit high brittleness, low impact resistance, and poor thermal stability as compared to petroleum-based polymers [51].

Different studies have incorporated antimicrobials into PHAs to obtain active food packaging materials (Table 3).

Basnett et al. [52] obtained PHA films with lime oil (5 wt% in the polymer solution) that were effective against *S. aureus*, even after one year of preparation. In contrast, these films did not inhibit the growth of gram-negative bacteria such as *E. coli*. Castro-Mayorga et al. [53] developed active antimicrobial suspensions in situ and by physical mixing (mix) of stabilized silver nanoparticles (0.5 mM, 1 mM and 2 mM). The antimicrobial effect of the film against *Listeria monocytogenes* was not effective after 24 h of exposure, but it markedly reduced the growth of *Salmonella enterica*, consistently with the described greater susceptibility of gram-negative bacteria. Xu et al. [54] developed PHA films with graphene oxide nanocomposites and long alkyl chain quaternary salt functionalized graphene oxide at 1, 3, 5 and 7 wt%. which showed 99.9% effectiveness against gram-negative and gram-positive bacteria. Xavier et al. [55] produced PHB from *Bacillus mycoides*, isolated from garden soil, and prepared antimicrobial films with the resulting PHB and vanillin (20, 40, 50, 80, 100, or 200 μg per gram of PHB) by solvent casting. Films were tested against *E. coli*, *S. typhimurium*, *S. flexneri* and *S. aureus* and results showed that the minimum vanillin concentration to reduce the microbial activity was 80 μg/g PHB. Correa et al. [56] developed blend nanocomposite films of PHB/PCL, organo-clays (Cloisite^®^ 30 B and 10A) and nisin. The organic clays exerted antimicrobial activity against *Lactobacillus plantarum* CRL691 although their inclusion in the polymer blend did not lead to antimicrobial films. The presence of clays did not affect the adsorption kinetics of nisin on PHB/PCL films. The PHB/PCL nisin-activated film was effective against *L. plantarum* (used as a model of processed meat spoilage bacteria) inoculated into sliced ham samples, thus extending its shelf-life. Narayanan et al. [57] prepared PHB-based antimicrobial films incorporating eugenol (10, 20, 40, 80, 100 and 200 μg/g polymer), and its antimicrobial activity against foodborne pathogens, spoilage bacteria and fungi was evaluated. The synergistic antimicrobial activity of the films was also investigated in the presence of crude pediocin. The culture broth containing pediocin, as well as the PHB antimicrobial film, showed a prolonged lag phase and a significant reduction in bacterial growth at 24 h. The culture broth with pediocin and eugenol incorporated into the PHB film worked synergistically. Fan et al. [58] developed active films of PHB with phosphoserine phosphatase (PSPH) (2 wt% of PSPH based on weight of PHB) by electrospinning and a bleached chlorine treatment, these films showed biocidal efficacy against *Staphylococcus aureus* (92.10% inhibition) and *Escherichia coli* (85.04%) in *in vitro* tests.

Figueroa-Lopez et al. [59] developed PHBV films using electrospinning, incorporating oregano oil (at 10 wt% in relation to the polymer) and zinc oxide nanoparticles (1, 3, 6 and 10 wt% of ZnONPs in PHBV). Both antimicrobials demonstrated their efficacy against *E. coli* and *S. aureus*, which was reduced after 15 days. The films containing a mixtures of both antimicrobials (2.5 wt% OEO + 2.25 wt% ZnONPs) showed the best results, since the activity was maintained for longer time periods, being the gram-positive bacteria the most susceptible to the antimicrobial effect. Requena et al. [63] developed antimicrobial PHBV films with carvacrol, eugenol, oregano or clove essential oil (13% *w*/*w*, in the film) that were sprayed between two layers of PHBV. For *L. innocua*, the most effective antimicrobial agent was carvacrol followed by oregano essential oil whereas clove oil or eugenol were less effective. The antibacterial effect of PHBV films with oregano or clove essential oil, or their main compounds, carvacrol and eugenol, respectively, was analyzed in different food matrices (cheese, chicken breast, pumpkin and melon) and *in vitro* tests with *Escherichia coli* and *Listeria innocua* were also performed. The reported antimicrobial activity in foods was less remarkable than that detected in the *in vitro* tests. No antilisterial effect was observed in the evaluated food matrices. The most significant antibacterial action against *E. coli* was observed in cheese and pumpkin samples, whereas the highest migration of both compounds took place in melon slices. A lack of correlation between the antibacterial effect and the active compound migration to the food matrix was observed, which suggested that many compositional factors affect the availability of the active compound to exert its antibacterial action in a specific food [63]. The same authors also obtained active bilayer films with PLA:PHBV (75:25) blend with carvacrol (25 g carvacrol/100 g polymer matrix) and starch sheets in a previous study [64]. These active bilayers inhibited the growth of *L. innocua* and *E. coli* from both contact sides of bilayers, depending on carvacrol internal diffusion through the film and subsequent release into the culture medium. Likewise, *E. coli* showed greater sensitivity than *Listeria* to all of antimicrobials tested.

Meléndez-Rodríguez et al. [61] developed antimicrobial PHBV films with a mesoporous support of silica and essential oil with eugenol that were evaluated in *in vitro* tests with *E. coli* and *S. aureus*. Microbial activity was reduced while the best efficiency was obtained for *S. aureus*. The eugenol-containing nanoparticles were loaded in the 2.5–20 wt% range into PHBV by electrospinning. Sabharwal et al. [62] incorporated triclosan (0.2, 0.4, 0.6, 0.8 and 1 g *w*/*w*) to PHBV films obtained by casting that were very effective against *Escherichia coli* and *Staphylococcus aureus*.

## 3. Polymer Biodegradation Studies in Different Media

**Figure 2:**
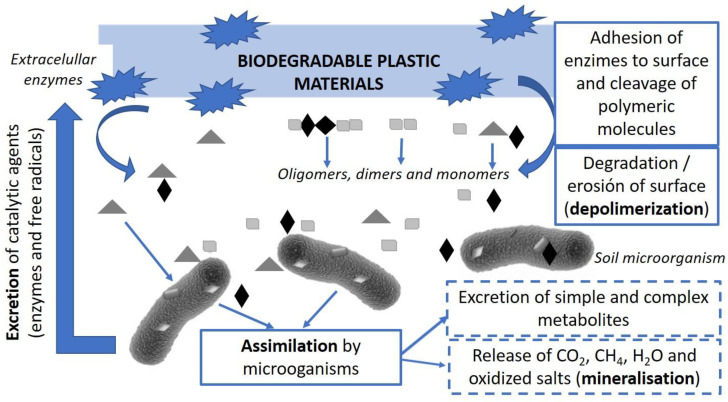
Polymer biodegradation pathway (Adapted from Youssef & El Sayed [67] and Lucas et al. [65]).

Polymer biodegradation involves biological activity and comprises of three main stages as seen in Figure 2: (1) biodeterioration or modification of the chemical, physical and mechanical properties of the polymer due to the growth of microorganisms on the surface of the material, (2) biofragmentation or conversion of polymers into oligomers or monomers by the action of the enzymes of microorganisms and (3) assimilation of the resulting compounds by microorganisms, as a source of carbon, energy and nutrients, and its conversion into CO_2_, water and biomass [65]. Some studies have been conducted to investigate the biodegradability of bioplastics under different environmental conditions, such as soil, compost, marine, and other aquatic environments [66]. Although most of the plastic waste is present in landfills, the biodegradation of plastics in landfills has not been significantly studied.

Composting and recycling are the two most widely considered procedures for managing plastic waste. Composting is a process in which organic matter is converted into CO_2_ and a soil-like material (humus) by the activity of a mixed group of microorganisms under controlled conditions [68]. This process allows to transform organic waste and by-products into quality materials used as soil improvers and/or fertilizers. In this way, the environmental impact that this waste generates is eliminated and the use of the abundant resources that they often contain is made possible. Then, biodegradation studies in compost media have been more extensively studied than in other media.

As defined by the American Society for Testing and Materials (ASTM), a compostable plastic undergoes degradation by biological processes during composting to produce CO_2_, water, inorganic compounds, and biomass at a rate consistent with other known compostable materials and leaves no visually-distinguishable or toxic residue. Therefore, a compostable plastic is biodegradable, while a biodegradable plastic is not always compostable [68].

Since plastic waste is also present in soil or aquatic environments, it is important to know the degradation behavior in these different media. Soil habitat contains a great biodiversity of microorganisms, which allows plastic biodegradation to be more feasible as compared to other environments, such as water or air [69]. On the other hand, plastic debris accumulates to a great extent uniformly in the marine environment. Due to its semi-permanent stability in marine ecosystems, plastic debris causes marine pollution, which has an impact on marine animals [70]. However, fewer studies have been conducted on the biodegradable potential of bioplastics in soil or aquatic environments. Furthermore, given the global accumulation of plastic waste in rivers, lakes, coastal waters and sediments, polar waters and deep waters, there is a need for more experimental data on polymer biodegradation in most aquatic ecosystems [71].

Factors that affect biodegradation processes include the chemical structure of the polymer, the type of chain and molecular complexity, the degree of crystallinity, and the enzymes secreted by microorganisms that act specifically on certain types of bonds. In general, polymers with a shorter chain, more amorphous and with less molecular complexity are more sensitive to the action of microbial enzymes. Likewise, the characteristics of the environment where the process takes place notably affect to biodegradation processes; humidity, temperature, pH, and oxygenation conditions are the most relevant factors for the microbial action responsible for the process. The optimal values or intervals of each parameter are influenced by the environmental conditions, the type of waste to be treated and the composting system. Biodegradation studies of polymeric materials have been carried out mainly in compost medium, basically, through the control of the amount of CO_2_ generated in the system because of the biodegradative action and mass loss of the samples due to the disintegration of the material, as a function of time. For the proper comparison of the behavior of different materials, standardized methods have been defined for carrying out the experiments under controlled conditions [72].

The moisture of the materials is considered the most important variable in composting [73] since the presence of water is essential for the physiological needs of the microorganisms. To ensure a good circulation of oxygen and other gases produced in the reaction, the humidity of the composting mass must be the required so that the water does not fully occupy the pores of the composting mass [74]. Optimal humidity for microbial growth is between 50–70% while temperature, pH, and oxygenation also play an important role. The increase in temperature of the composting mass is the clearest indication of the existence of microbial activity [74]. In the aerobic decomposition process an initial mesophilic phase (T < 45 °C) and a final thermophilic phase (T > 45 °C) can be distinguished, considering the process finished when the initial temperature is reached again. pH monitoring throughout the process allows for obtaining an indirect measurement of the aeration control of the mixture, and the existing relationships between pH-aeration-microorganisms, since the organic degradation process is inhibited at low pH. A pH that remains above 7.5 during the process, indicates a good decomposition [75]. Several authors divide the evolution of composting into three phases: (1) the initial mesophilic phase, where there is a decrease in pH due to the action of microorganisms on the most labile organic matter, releasing organic acids; (2) a second phase where there is a gradual alkalinization of the medium, and (3) the third phase where the pH tends to neutrality due to the formation of humic compounds [76]. During the composting process it is necessary to ensure the presence of oxygen and avoid insufficient aeration since it would cause the substitution of aerobic microorganisms by anaerobes, retarding decomposition and giving rise to the appearance of bad odors and compounds such as hydrogen sulfide [77].

Table 4 summarizes the values of the percentage and time of biodegradation of different bioplastics in different media and conditions reported by several authors, where the effect of the environmental conditions, such as average pH, moisture and oxygen contents, and temperature on the polymer biodegradation behavior, can be observed.

The structure and composition of the biomaterials extremely affect the biodegradation process in its different stages [93]. In this sense, the range of values observed in Table 4 for PLA is attributed to the effect that different fillers used have on biodegradation behavior [69]. In general, it has been observed that those fillers enhancing the hydrophilicity of the composite material promote the polymer hydration capacity and the effectiveness of the degradative action of microorganisms. In contrast, the increase in the material hydrophobicity decreases the rate of biodegradation, Likewise, those additives or mechanisms that inhibit the crystallization of the polymer promote degradation rate. PHBV, where the copolymerization with hydroxyvalerate decreases the crystallinity with respect to the PHB, exhibited a more effective biodegradation than PHB [94]. However, most biodegradation studies have been carried out on pure biopolymers or mixtures, without taking into account the different additives that are added to enhance the functionality of the material. The addition of plasticizers, many of them non-biodegradable, increases the longevity of the bioplastic in the environment. In general, degradation in a compost medium is more effective than in soil or aquatic environments due to the richness of the active microbial population and the ability to adjust environmental conditions. In marine environments, different marine habitats with very different biodegradation conditions must be considered [95]. Thus, in deep waters, the lack of UV radiation and low temperatures and O_2_ concentration make the biodegradation process slower, being the extensive degradation less likely than in other environments [96]. For this reason, some authors have suggested the need of biodegradation studies in six different aquatic habitats: supralittoral (splash zone), eulittoral (or intertidal), sub-coastal (subtidal), benthic region (marine), pelagic and buried in sediments. These habitats mainly differ in temperature, UV light, pressure, density and oxygen and nutrient content. Thus, different authors have found that biodegradation in pelagic habitat (near surface seawater) is more efficient than in eutrophic habitat (lakes and reservoirs with excess phytoplankton) and that the maximum rate of biodegradation occur at the water-sediment interface for being richer in microorganisms [95].

The flow conditions of the water mass (static or dynamic) also affected the biodegradation pattern. Some studies showed that the biodegradation rate is higher under static than dynamic conditions surely because of the broader temperature changes and the limited supply of nutrients under dynamic conditions. Likewise, when plastics are buried, in close contact with sediments, the biodegradation is usually positively influenced [85].

Although in aquatic systems temperature presents great variations depending on the depth, the season of the year and the geographical area, most of the studies shown in Table 4 were carried out between 12–22 °C (pH between 7.9 and 8.1). Among the microorganisms capable of degrading plastics in aquatic systems (marine and freshwater), *Pseudomonas*, Bacillus, *Alvanivorax*, *Tenacibaculum*, *Lepthotrix*, *Enterobacter*, *Variovorax* and *Actinomyces*, including *Streptomyces,* have been found. Volova et al. [70] evaluated the effect of the shape/3D dimension of the polymeric material, concluding that PHA films degraded faster than PHA pellets due to their greater surface area, which facilitates the adhesion of microorganisms to their surface, this being beneficial for the polymer degradation.

As can be observed in Table 4, most of the studies found in aquatic systems do not evaluate the conversion of polymer carbon into CO_2_ by microorganisms, but rather use other techniques that indirectly allows for estimating the total degradation of the plastic (carried out in a biotic and abiotic processes). In this way, these studies were carried out by measuring the changes in the material physicochemical properties, such as mechanical, molecular weight or mass loss, which became altered because of the degradative process. Thus, these can be not strictly considered as biodegradation but degradation studies.

## 4. Effect of Antimicrobials on the Biodegradation of Polymer Based Active Packaging Materials

Different studies have analyzed the influence of antimicrobials, on the biodegradation behavior of active packaging materials, as shown in Table 5. The viability and growth of relevant microorganisms that are involved in the biodegradation process can be affected by the released antimicrobial compounds after swelling or disintegration of the polymeric matrix. The action mechanism that takes place in the composting medium will depend on the nature of the antimicrobial (volatility, level of persistence in the medium, etc.), and coincides with the mechanisms that confers the antimicrobial action to the films. Usual action mechanisms include the alteration of the cell permeability leading to disrupted bacterial cell membranes and releasing the cellular content.

Sen & Das [97] observed 90% biodegradation in soil of PVA films formulated with starch and sodium propionate (1.791 g/100 g of polymer) throughout 28 days. In this study, the antimicrobial compound did not interfere with the action of the soil microflora and did not hinder the biodegradation of the film, which simultaneously contributed to the increase of nutrients in the soil. Furthermore, the pH was kept within the accepted limit for plant growth. Therefore, the presence of sodium propionate in the film did not prevent their biodegradation, and the films exerted a positive effect on plant growth. Ulloa et al. [98] carried out a biodegradability test in soil, with PLA films containing propolis at different concentrations: powdered raw propolis and ethanolic extract of propolis at concentrations of 5, 8.5, and 13% *w*/*w* of PLA. The authors observed a higher weight loss in the films incorporating active compounds, with values ranging between 2.5 and 5% for films with crude propolis powder and 9–24% after 314 days for films with ethanolic extract of propolis. The presence of propolis supposed a contribution of nutrients for the microorganisms, thus enhancing film degradation.

Costa et al. [99] studied the biodegradation of PHBV with silver nanoparticles (Ag/PHBV was 0.024 wt%) in a tropical soil under laboratory conditions incorporating Biochar^®^n (charcoal obtained from plant debris and waste biomass of sugarcane bagasse). Biochar^®^ was used as a tool to accelerate the compound degradation. The addition of 5–10% Biochar^®^ in the soil increased the degradation of these polymeric materials 2 to 3 times after 30 days of incubation ground. However, the presence of silver nanoparticles significantly reduced the potential for degradability of the nanocomposite by the microbial community of the soil.

Pavoni et al. [100] studied the biodegradation of starch/chitosan blend films. The mixture of starch:chitosan solutions were prepared using different proportions of 1:1, 2:1 and 3:1 (*v*/*v*). Incorporating chitosan did not interfere with the behavior of biodegradation under the conditions considered in compost medium. However, Hasan et al. [16] concluded that the biodegradation rate of the starch/chitosan films (at 30:70, 50:50 and 70:30 starch/chitosan ratios) was strongly influenced by the content of chitosan; the greater the chitosan content the lower the biodegradation rate of the films in the composting medium, thus reflecting the antimicrobial effect of chitosan on the compost population.

Cano et al. [101] obtained films of starch, PVA and mixtures of both polymers with different concentrations of neem oil, oregano essential oil (1:0.5 and 1:0.125 starch:oil ratio) and silver nanoparticles at different weight ratios with respect to starch (1:0.006, 1:0.06, 1:0.16 and 1:0.32). The analysis of their decay behavior and biodegradation in compost medium during 73 and 45 days showed that the presence of neem and oregano oils improved disintegration levels and biodegradation. However, the biodegradability of films incorporating silver nanoparticles was seriously diminished, reflecting the influence of silver nanoparticles on the activity of the microbial population of the compost as silver lasts longer in the environment in comparison with the essential oils. Wang et al. [102] investigated the biodegradation behavior of PBAT (poly(butylene adipate-co-terephthalate) films and thermoplastic starch. The decay data (loss weight) showed that both PBAT and starch could be degraded, even with the presence of the antimicrobial substances (polyhexamethylene guanidine hydrochloride) at 1.0 and 0.99 wt%, but antimicrobials reduced the biodegradation rate.

Norcino et al. [103] incorporated copaiba oil nano-emulsion as an antimicrobial in pectin films that were prepared from aqueous solutions (6% wt.) of pectin combined at 1:1 (weight ratio) with the previously prepared nanoemulsions (1–6% *w*/*w*). Reported results showed a gradual decrease in the production of CO_2_ as the concentration of the antimicrobial increased, thus indicating that the active compounds of the copaiba oil nano-emulsion interfered with the biodegradation pattern of the pectin films in soil. Tampau et al. [104] evaluated the biodegradation behavior of multilayer films of thermoplastic starch and PCL, with carvacrol (15 g carvacrol/100 g PCL) incorporated into the PCL layer, through weight loss and CO_2_ measurements. All carvacrol-free films completely biodegraded after 25 days of composting. However, the presence of carvacrol notably affected the activity of the inoculum, thus limiting the biodegradability of the carvacrol loaded multilayers to a maximum value of about 85% after 45 days. Arrieta et al. [105] obtained various bilayer formulations of PHBV/PLA with catechin (1 and 3 wt%) and lactic acid oligomers. After 23 days of disintegration in compost, the bilayer systems began to separate. The incorporation of catechin slightly delayed the disintegration process, while lactic acid accelerated it. Tang et al. [106] prepared several starch/PVA/nano-SiO_2_ (0.5, 0.10, 0.15, 0.20, 0.25, 0.30, 0.35, 0.40, 0.45 and 0.5 g SiO_2_/10 g starch/PVA) blend films by casting. The soil burial test showed that the addition of nano-SiO_2_ did not have a significant influence on biodegradability of the films. Capelezzo et al. [107] evaluated the biodegradation in soil of Ecoflex^®^ polymers with zinc oxide nanoparticles (1 and 2% *w*/*w*), as an antimicrobial. The addition of zinc compounds to the biodegradable polymer did not affect its behavior biodegradation. Lyu et al. [45] developed PCL films incorporating different concentrations of grapefruit seed extract (GSE: 0, 1, 3 and 5 %) as antimicrobial agent and evaluated the biodegradation in soil. Biodegradation was faster as the amount of antimicrobial incorporated increased, in line with the lower cohesiveness of the PCL matrix when active compound was present and the lack of effective interferences of GSE with the microbial population activity.

In most of studies, no great effect of the incorporated antimicrobials on film degradation was observed, although in some cases a delay of the process was caused by the influence of active compounds on the microbial population and activity of the medium. The potential changes in the biodegradation behavior of the polymer in the antimicrobial materials will depend on the doses of the active compounds in the films, their release kinetics to the medium, and the sensitivity of the different microorganism responsible for the degradative process. Moreover, the incorporation of antimicrobials modifies the physical structure of the matrix, their permeability properties and wetting capacity. This can favor, in some cases, its disintegration and biodegradation process. When the active compounds promote the hydrophilic nature of the polymeric matrix and so, its wetting capacity, its sensitivity to the microbial action is enhanced. No biodegradation studies of antimicrobial films have been found in aquatic environments. Therefore, specific studies are necessary in each case to know the influence of a determined antimicrobial on the degradation behavior of a specific polymeric matrix in different media.

## 5. Final Remarks

Biodegradation of different bioplastics (from biomass, synthetic or produced by microorganisms) depends on the polymer molecular structure, crystallinity and fillers or incorporated compounds, as well as on the environmental conditions, such as microorganism population, pH, humidity and temperature of the compost, soil or aquatic media. Antimicrobial materials have been obtained from these biopolymers, by incorporating active compounds, such as plant extracts, essential oils or their compounds and inorganic compounds, such as silver or zinc nanoparticles. These materials have high potential for food packaging since they allow to extend the product shelf-life but can negatively affect the material biodegradation in determined environmental conditions. More biodegradation studies in different media (compost, soil, and marine environments) are needed to ensure the safety of these materials exposed to different ecosystems. Composting is a sustainable strategy for the management of these biopolymer-based active packaging materials, but it is necessary to ensure that the effect of the incorporated antimicrobials does not affect the process. On the other hand, the biodegradation of bioplastics in aquatic and marine ecosystems requires in-depth studies since the accumulation of bioplastics can inevitably occur in these environments.

## Figures and Tables

**Table 1:** Different studies on antimicrobial starch-based films.

Biodegradable Polymer	Antimicrobial	Microbiological Tests	Microorganisms	Results	Reference
Cassava starch/cellulose nanofibers	Tea tree essential oil	*In vitro*	*E. coli* *S. aureus* *C. albicans*	*S. aureus*: inhibition of 73% *C. albicans*: inhibition of 65% *E. coli*: no effect	[13]
Cassava starch	Cinnamon essential oil	*In vitro*	*P. commune* *E. amstelodami*	Mayor inhibition of *E. amstelodami*	[14]
Pea starch/PVA	Silver nanoparticles	*In vitro*	*L. innocua* *E. coli* *A. niger* *P. expansum*	Microbial growth inhibition	[15]
Brown rice starch /chitosan	Chitosan	*In vitro*	*E. coli* *S. aureus*	Microbial growth inhibition	[16]
Cassava starch/chitosan	Oregano and cinnamon leaf essential oils	Pork meat	Total aerobic and coliform	No growth inhibition	[10]
Cassava starch/chitosan	Chitosan	Pork meat	Total aerobic and coliform	Microbial growth inhibition	[11]
Sugar palm starch/nanocrystalline celulose	Cinnamon essential oil	*In vitro*	*B. susbtilis* *S. aureus* *E. coli*	Microbial growth inhibition	[17]
Tapioca starch	Chitosan	*In vitro/Cherry tomato*	*B. cereus* *S. aureus E. coli*, *S. typhimurium*	Microbial growth inhibition	[18]
Corn starch/bovine gelatin	N-α-lauroyl-l-arginine ethyl ester monohydrochloride	Chicken breast	*Psychotrophic bacteria, lactic acid bacteria, anaerobic, total coliforms, E.coli*	Microbial growth inhibition	[12]
Pea starch/PVA	Neem and oregano essential oils	*In vitro*	*L. innocua* and *E. coli*	Microbial growth inhibition	[19]
Cassava starch/chitosan	Lemongrass essential oil	*In vitro*	Mesophillic bacteria	Microbial growth inhibition	[20]
Sago starch/guar gum	Carvacrol and citral	*In vitro*	*B. cereus* *E. coli*	Microbial growth inhibition	[21]
Oxidized and Acetylated Corn Starch / Sodium Alginate	Sodium dehydroacetate and rosemary extract	*In vitro*	*E. coli* *A. niger*	Microbial growth inhibition	[22]
Hydroxypropyl-high-amylose starch	Pomegranate peel powder	*In vitro*	*S. aureus* *Salmonella*	Greater action against *S. aureus* than against *Salmonella*	[23]
Antimicrobial starch	Sodium benzoate and citric acid	Cheddar cheese	*L. innocua*	Microbial growth inhibition	[24]
Corn starch/bovine gelatine	Ethyl lauroyl arginate (LAE)	Marinated salmon	*L. innocua*	Microbial growth inhibition	[25]

**Table 2:** Different studies on antimicrobial films based on synthetic biodegradable polymers (PLA, PVA and PCL).

Biodegradable Polymer	Antimicrobial	Microbiological Tests	Microorganisms	Results	Reference
PLA	Essential oils (clove, cinnamon and garlic)	*In vitro*	*C. jejuni* *S. aureus*	Garlic oil: limited antimicrobial activity Clove and cinnamon oils: more effective against *C. jejuni* than against *S. aureus*	[32]
PLA/PBAT	Cellulose-silver nanocrystals	*In vitro*	*E. coli* *S. Aureus*	Limited antimicrobial activity	[33]
PLA/PBS	Chitin nanofrils	*In vitro*	*S. aureus* *Enterobacter* spp.	No microbial inhibition	[34]
PLA	Propolis ethanolic extract	*In vitro*	*Gram positivas* *Gram negativas*	No efective	[35]
PLA	Propolis ethanolic extract Essential oil of Tanacetum balsamita	*In vitro*	*B. cereus* *Gram positive* *Gram negative*	Limited antimicrobial activity	[35]
PLA	Propolis ethanolic extract Tanacetum balsamita essential oil	Sausages	*Lactic acid, aerobic esophilic and psychotrophic bacterias*	Extended shelf-life of sausages	[35]
PLA/Starch	Cinnamaldehyde	*In vitro*	*E. coli* *L. innocua*	Inhibition of microbial growth	[36]
PLA	Chitosan	Pork meat	*Total aerobes and coliform*	Inhibition of microbial growth	[37]
PVA	poly (hexamethylene guanidine)	*In vitro*	*S. aureus* *E. coli*	Inhibition of microbial growth	[38]
PVA-Chitosan	Ethyl Lauroyl Arginate (LAE)	*In vitro*	*C. jejuni*, *S. typhimurium E.coli*, *L. monocytogenes*	Inhibition of microbial growth with 5–10% LAE	[39]
PVA	Lactic, tartaric and malic acids	*In vitro*	*S. aureus* *E. coli*	Greater inhibition with lactic acid, followed by malic and tartaric acids	[40]
PVA-Chitosan	Chitosan	Minimally processed tomato	*S. aureus* *E. coli* *B. subtilis*	Greater inhibitory effect in *E. coli* and *B. subtilis* than in *S. aureus*	[41]
PCL/short chain peptide (REDV)	Eugenol	*In vitro*	*E. coli* *S. aureus*	Limited antimicrobial activity	[42]
PCL	Solid extract of sage	*In vitro*	*E. coli* *S. aureus*	Limited antimicrobial activity. More effective against *S. aureus*	[43]
PCL	Organic acids, rosmarinic acid extract and Asian essential oil blend.	Broccoli	*E. coli* *S. typhimurium*	Inhibition of microbial growth	[44]
PCL	Grapefruit seed extract	*In vitro*	*L. monocytogenes*	Inhibition of microbial growth	[45]
PCL/starch	Pomegranate rind powder	*In vitro*	*S. aureus*	Inhibition of microbial growth at high concentrations	[46]
PCL	Cinnamaldehyde	*In vitro*	*E. coli* *S. aureus*	Inhibition of microbial growth	[47]
PCL	Methanolic extract of pomegranate	*In vitro*	*E. coli* *S. aureus*	6–7 days growth delay	[47]
PCL	Freeze dried pomegranate arils	*In vitro*	*E. coli* *S. aureus*	2-day growth delay	[47]
PCL	Pomegranate seed flour	*In vitro*	*E. coli* *S. aureus*	2-day growth delay	[47]
PCL/starch	Carvacrol	*In vitro*	*E. coli* *L. innoua*	Inhibition of *E.coli*	[48]

**Table 3:** Different studies on antimicrobial films based on biopolymers obtained from microorganisms (PHA, PHB and PHBV).

Biodegradable Polymer	Antimicrobial	Microbiological Tests	Microorganisms	Results	Reference
PHA	Lime oil	*In vitro*	*E. coli* *S. aureus*	Antimicrobial effectiveness against *S. aureus*	[52]
PHBV	Silver nanoparticles	*In vitro*	*S. enterica* *L. monocytogenes*	Effective against *S.enterica* and not effective against *L. monocytogenes*	[53]
PHA	Alkyl quaternary ammonium salts	*In vitro*	*E. coli* *S. aureus*	Inhibition of microbial growth	[54]
PHB	Vanillin	*In vitro*	*E. coli* *S. typhimurium* *S. flexneri* *S. aureus*	Minimum concentration to reduce microbial activity: 80 μg/g PHB	[55]
PHB/PCL/Organic clays	Nisin	*In vitro*/ham slices	*L. plantarum*	Inhibition of microbial growth	[56]
PHB	Eugenol and pediocin	*In vitro*	*S.aureus* *E. coli* *S. typhimurium* *B. cereus*	Inhibition of microbial growth	[57]
PHB/PSPH	Chlorine	*In vitro*	*S.aureus* *E. coli*	Inhibition of microbial growth	[58]
PHBV	ZnO nanoparticles and oregano essential oil	*In vitro*	*E. coli* *S. aureus*	Significant microbial growth inhibition	[59]
PHBV	ZnO nanoparticles and oregano essential oil acting synergistically	*In vitro*	*E. coli* *S. aureus*	Greater microbial inhibition than that of pure antimicrobials	[59]
PHBV	Oregano essential oil	*In vitro*	*E. coli* *L. innocua*	Significant microbial inhibition	[60]
PHBV	Carvacrol	*In vitro*	*E. coli* *L. innocua*	Significant microbial inhibition	[60]
PHBV/Silica mesoporous support	Eugenol essential oil	*In vitro*	*E. coli* *S. aureus*	Microbial inhibition	[61]
PHBV	Triclosan	*In vitro*	*E. coli* *S. aureus*	Effective microbial inhibition	[62]
PHBV	Carvacrol/Eugenol	*In vitro*	*E.coli* *L. innocua*	Effective microbial inhibition	[63]

AgNP: silver nanoparticles, PSPH: phosphoserine phosphatase.

**Table 4:** Biodegradation studies of some bioplastics in compost, soil or aquatic environments.

Bioplastics	Type of Environment	Conditions	Control Method	Biodegradation Period (days)	Biodegradation (%) Others Data	Reference
**PLA**						
PLA	Compost	58 °C	Produced CO_2_	80	78.9	[78]
PLA/TiO_2_ nanocomposites	Compost	58 °C	Produced CO_2_	80	Between 85 and 97.8	[78]
PLA	Compost	58 °C, 50% humidity	Weight loss	14	>90	[79]
PLA/CNC nanocomposites	Compost	58 °C, 50% humidity	Weight loss	14	>90	[79]
PLA	Soil	-	Weight loss	70	0.15	[80]
PLA/Starch	Soil	-	Weight loss	70	16	[80]
PLA	Sea water and freshwater	25 °C and fluorescence light (16 h light and 8 h dark)	Weight loss	365	Non-significant degradation	[81]
PLA	Compost	58 °C	Produced CO_2_	130	90	[82]
PLA	Sea water	Without sediment, in euphotic and aphotic conditions	Weight loss	365	PLA > PET	[83]
PLGA	Sea water and fresh water	25 °C and fluorescence light (16 h light and 8 h dark)	Weight loss	270	100	[81]
			**PHAs**			
3-PHB	Sea water	28.75 ± 1.65 °C 53% salinity pH 7.0–7.5	Weight loss	160 (films) 80–160 (pellets)	58 (films) 38 (pellets)	[70]
PHB/PHBV	River water	Eutrophic recreation. (1 m depth)	Weight loss Degradation rate (DR)	31–42	34.6–43.5% DR: 0.011–0.014 d^−1^	[84]
PHB	River water	Eutrophic recreation (1 m depth)	Weight loss Degradation rate (DR)	22–45	93% DR: 0.008–0.174 d^−1^	[84]
PHB	Sea water and fresh water	25 °C and fluorescence light (16 h light and 8 h dark)	Weight loss	365	8.5	[81]
3-PHB/3-PHV	Sea water	28.75 ± 1.65 °C 53% salinity pH 7.0–7.5	Weight loss	160 (films) 80–160 (pellets)	54 (films) (13 pellets)	[70]
PHBV	Sea water	Laboratory (static), 30 °C. (With sediment, 75 mL) Aquarium (dynamic): 12–22 °C. With and without sediment.	Produced CO_2_ and Weight loss (WL)	38–90	% CO_2_: 70% WL static: 75–85% WL dynamic: 33–50%	[85]
PHB	Sea water	Laboratory (static): 30 °C. (With sediment, 75 mL) Aquarium (dynamic): 12–22 °C. With and without sediment.	Produced CO_2_ and weight loss (WL)	18–100	% CO_2_: 80–90% WL static: 90% WL dynamic: <90%	[85]
PHB	Sea water	Intertidal zone, pelagic (10 m depth), benthic (20 m depth).	Degradation rate (DR)	-	DR: Benthic > Intertidal > Pelagic	[86]
PHBV	Soil	-	Weight loss	112	0.5	[87]
PHBV flax	Soil	-	Weight loss	112	6	[87]
PHBV/PBAT/ flax	Soil	-	Weight loss	112	9	[87]
PHBV/ENR flax	Soil	-	Weight loss	112	17	[87]
PHBV	Compost	58 °C	Produced CO_2_	100	63.2	[88]
PHBV/flaxseed fibers	Compost	58 °C	Produced CO_2_	100	85.6	[88]
PHBV/flax/ alginic	Compost	58 °C	Produced CO_2_	100	88.0	[88]
PHBHHx/PBAT	Sea water	-	Weight loss	28	31 (ratio 100/0) 19 (ratio 80/20) 10 (ratio 60/40) 3 (ratio 40/60) 1 (ratio 0/100)	[89]
PHBHHx/PBS	Sea water	-	Weight loss	28	51 (ratio 100/0) 41(ratio 80/20) 18 (ratio 60/40) 5 (ratio 40/60) 1 (ratio 0/100)	[89]
PHBHHx/PLA	Sea water	-	Weight loss	28	34 (ratio 100/0) 33 (ratio 80/20) 32 (ratio 60/40) 26 (ratio 40/60) 1 (ratio 0/100)	[89]
			**PCL**			
PCL	Compost	58 °C	Produced CO_2_	72	~100	[90]
PCL/TPS	Compost	58 °C	Produced CO_2_	72	~90 (ratio 50/50) ~95 (ratio 30/70)	[90]
PCL	Soil	30 °C	Weight loss	90	2.5	[45]
PCL	Sea water	Depth: 321 m, 350 m, 612 m. Low temperatures and high hydrostatic pressure.	Resistance to break, (RB) and surface morphology (SM)	270–360	RB decrease: 0–20% SM: abundant pores and heterogeneous cracks	[91]
PCL	Sea water and fresh water	25 °C and fluorescence light (16 h light and 8 h dark)	Weight loss	365	Non-significant degradability	[81]
			Others			
PBS/Starch	Soil	25 °C, 60% humidity	Weight loss	28	7 (films) 24 (powdered)	[92]
PBS	Soil	25 °C, 60% humidity	Weight loss	28	1 (films) 16.8 (powdered)	[92]
PVA	Compost	-	Iodometric analysis	8	51–79	[23]
PBS	Sea water	Depth: 321 m, 350 m, 612 m. Low temperatures and high hydrostatic pressure.	Resistance to break, RB) and surface morphology (SM)	360	RB decrease ≈ 100% SM: rough surface with many stains	[91]
PBSe	Sea water	Intertidal zone, pelagic (10 m depth), benthic (20 m depth).	Weight loss and Degradation rate (DR)	-	DT: Benthic > Intertidal > Pelagic	[86]
PBSet	Sea water	Intertidal zone, pelagic (10 m depth), benthic (20 m depth).	Weight loss Degradation rate (DR)	-	DT: Benthic > Intertidal > Pelagic	[86]

CNC: cellulose nanocrystal, ENR: epoxidized natural rubber, PBSe: Polybutylene sebacate, PBSet: Polybutylene sebacate-co-terephthalate, PHBHHx: poly(3-hydroxybutyrate-*co*-3-hydroxyhexanoate), PHV: polyhydroxyvalerate, PLGA: poly(lactic-co-glycolic acid), TPS: thermoplastic starch.

**Table 5:** Some effects of the incorporated antimicrobial compounds on the biodegradation of polymeric films.

Polymer	Antimicrobial	Type of Environment	Main Feature	Reference
Starch/PVA	Sodium propionate	Soil	The antimicrobial did not interfere with biodegradation. 90% degradation in 28 days	[97]
PLA	Propolis (crude propolis and its ethanolic extract)	Soil	Propolis promoted biodegradation	[98]
PHBV	Silver nanoparticles	Soil	Biochar accelerated biodegradation. Silver nanoparticles significantly reduced biodegradability	[99]
Maize starch/chitosan	Chitosan	Compost	In 15 days, the chitosan did not negatively affect the biodegradation	[100]
Brown rice starch/chitosan	Chitosan	Compost	Biodegradation was faster with higher proportion of starch	[16]
Starch/PVA	Neem oil, oregano essential oil and silver nanoparticles	Compost	The oils improved the biodegradation of films Silver nanoparticles inhibited biodegradation	[101]
PBAT/ thermoplastic starch	Polyhexamethylene Guanidine Hydrochloride (PHPG)	Soil	Antimicrobial delayed the biodegradation	[102]
Pectin	Copaiba oil	Soil	Delay biodegradation of polymer	[103]
Starch/PCL	Carvacrol	Compost	Carvacrol delayed biodegradation	[104]
PHBV/PLA-PHB	Catechin	Compost	Catechin delayed disintegration process Lactic acid accelerated it	[105]
Starch/PVA	Silicon oxide nanoparticles	Soil	Silicon oxide nanoparticles did not affected biodegradation	[106]
Ecoflex^®^	Zinc oxide nanoparticles and microcapsules with ionic zinc	Soil	Zinc compounds did not affect biodegradation process	[107]
PCL	Grapefruit seed extract (GSE)	Soil	Biodegradation was faster as the incorporated amount of GSE increased	[45]
